# Genome-wide RAD sequencing to identify a sex-specific marker in Chinese giant salamander *Andrias davidianus*

**DOI:** 10.1186/s12864-019-5771-5

**Published:** 2019-05-23

**Authors:** Qiaomu Hu, Cuifang Chang, Quanhe Wang, Haifeng Tian, Zhigang Qiao, Lei Wang, Yan Meng, Cunshuan Xu, Hanbing Xiao

**Affiliations:** 1 Yangtze River Fisheries Research Institute, Chinese Academy of Fishery Sciences, Wuhan, 430223 Hubei China; 2 State Key Laboratory Cultivation Base for Cell Differentiation Regulation, College of Life Science, Henan Normal University, Xinxiang, 453007 Henan Province China; 3 College of Life Science, Yangtze University, Jingzhou, 434025 China; 4 College of Fisheries, Henan Normal University, Xinxiang, 453007 China

**Keywords:** *Andrias davidianus*, RAD-seq, Female-specific marker, Sex identification, Sex reversal

## Abstract

**Background:**

Chinese giant salamander *Andrias davidianus* is an endangered species. The success of artificial breeding provides a useful way to protect this species. However, the method to identify the sex and mechanism of sex determination were unclear which hinder the improvement of the artificial breeding. Detection of a sex specific marker provides an effective approach to identify genetic sex and investigate the sex determination mechanism.

**Results:**

We used restriction-site-associated DNA (RAD) sequencing to isolate a sex-specific genetic marker in *A. davidianus* to expand knowledge of the sex determination mechanism. Four male and four female specimens were subjected to RAD sequencing, which generated 934,072,989 reads containing approximately 134.4 Gb of sequences. The first round of comparison of the assembled sequence against the opposite sex raw reads revealed 19,097 female and 17,994 male unmatched sequences. Subsequently, 19,097 female sequences were subjected to a BLAST search against male genomic data, which revealed 308 sequences unmapped to the male genome. One hundred of these were randomly selected and validated by PCR in five male and five female specimens, and four putative sex-specific sequences were produced. Further validation was performed by PCR in another 24 females and 24 males, and all female individuals exhibited the expected specific bands, while the males did not. To apply the sex-specific marker, three specimens reversed from genetic female to physiological male were found in a group exposed to elevated temperature, and 13 individuals reversed from genetic male to physiological female were obtained in a 17β-estradiol exposed group.

**Conclusion:**

This is the first report of a sex-specific marker in *A. davidianus* and may have potential for elucidation of its sex determination mechanism and, hence, its conservation*.*

**Electronic supplementary material:**

The online version of this article (10.1186/s12864-019-5771-5) contains supplementary material, which is available to authorized users.

## Background

Genetic mechanisms, including sex determination, are highly variable among vertebrates. Genetic sex determination can be either male heterogamety (XX/XY) or female heterogamety (ZZ/ZW) [1, 2]. To understand the fundamental biological processes of sex determination and sex reversal, the identification of sex chromosomes is necessary. Sex manipulation in breeding made possible by identification of the sex chromosomes can have considerable economic value [3, 4].

Several techniques can be employed to identify chromosomal sex. Most simply, the karyotype is visualized by cytogenetic techniques to detect the heteromorphic sex chromosome [5–7]. Breeding of sex reversed neomales and neofemales can reveal which sex is heteromorphic based on the sex ratio of the progeny [8–12]. In toad *Bufo bufo*, a sex reversed female mated with the normal male produced all male progeny, indicating male homogametic sex determination mechanism (ZZ) and female heterogametic (ZW) [8]. Gynogenesis uses heterogenous sperm or inactivated sperm to activate eggs, which are then heat-shocked to prevent the second meiotic division, essentially producing female self-fertilization. This technique yields all female progeny, indicating the XX/XY system in the studied species [13–17].

Each of these techniques presents an associated challenge. The karyotype is not available for fish, amphibians, and reptiles that possess a telocentric chromosome or lack the heteromorphic sex chromosome [18, 19]. Both producing a sex reversed individual in many species as well as producing sufficient numbers of progeny to analyze the sex ratio to infer the genetic type are challenge. Development of a sex-specific marker shows excellent potential for identification of the sex chromosome in various species.

A sex-specific marker is especially valuable in species that lack distinguishable sexually dimorphic phenotypes and in specimens at early stages of development that lack secondary sex characteristics. The most common means of investigating a sex-specific marker involves amplified fragment length polymorphism (AFLP) [3, 20] or microsatellites [21, 22]. However, the effort involved in these methods is enormous and these approaches cannot be applied for all of the species. Additionally, if any restriction enzyme failed to identify a sex-specific marker, the AFLP can’t switch to another enzyme that cut more frequently in the genome. Recently, restriction-site-associated DNA sequencing (RAD-seq) has been employed to identify sex-specific markers and describe sex determination in many species [23–29]. In *Anolis carolinensis*, sex-specific molecular markers identified using RAD-seq were found to be conserved in other Anolis species [27]. In *Danio rerio,* RAD-seq was employed to analyze F2 offspring reciprocal crosses and revealed a sex-associated locus at the end of the arm of Chr-4 in both family A (offspring of Nadia female and *AB male http://zfin.org/action/genotype/genotype-detail?zdbID=ZDBFISH-960809-7) and family B (offspring of *AB female and a Nadia male) as well as a locus on chr-3 in family B [24].

Chinese giant salamander *Andrias davidianus* is the world’s largest extant amphibian and was historically widely distributed in China. However, due to the environmental degradation and human harvesting, the wild population has sharply decreased, and it is now classified as an endangered species. *A.davidianus* possesses 30 pairs of chromosomes with 19 pairs of microchromosomes [18] and a genome of ∼50 GB, making assembly a challenge [30]. Therefore, RAD-seq is a useful method to explore a sex-specific marker in *A. davidianus*.

The goal of this study was to identify a sex-specific genetic marker in *A. davidianus* through the RAD-seq. We firstly used the female RAD-seq data to check against the male RAD-seq data and produced the putative female specific sequences, and then the putative female specific sequences were blasted against the male genome data and produced the candidate sex specific sequences. The candidate sex specific marker was validated by comparing to individuals with sex identified by PCR amplification. These markers were used to identify the sex reversal salamander from the individuals exposed to high temperature or sex hormone.

## Methods

### RAD library construction and sequencing

Two male (X1, X2) and two female (C1, C2) one-year-old, apparently healthy, *A. davidianus* were obtained from Zhejiang Yongqiang Chinese Giant Salamander Ltd. (Jinhua, Zhejiang Province, China). An additional two male (X3, X8) and female (C3, C8) adult *A. davidianus* were obtained from Shandong Yimeng Chinese Giant Salamander Ltd. (Yimeng, Shandong Province, China) (Table 1). The individuals were killed after anesthesia with MS222 according to Yangtze River Fisheries Research Institute Care Committee (No. 2013001). Physiological sex was determined by histology. Genomic DNA was extracted using the TIANamp Genomic DNA Kit (Tiangen, Beijing, China) including RNase A treatment, and the concentration and quality was detected by Agilent 2100 Bioanalyzer (Agilent Technologies, Santa Clara, CA) and agarose gel electrophoresis. Genomic DNA was double-digested using restriction enzymes EcoRI and NlaIII (New England Biolabs) following the conditions 1 μl of EcoRI-HF (Fermantas, 20 units), 1 μl of NIAIII (Fermantas, 20 units), 5 μl of Fermantas buffer, 25 μl of DNA (500 ng of DNA), and double-distilled water to a final volume of 50 μl. The reaction was incubated at 37 °C for 1 h and subsequently at 65 °C for 30 min to inactivate the restriction enzyme. The resulting fragment was purified by MiniElut DNA-Pure Kit (Sangon Biotech, Shanghai, China) and ligated to P1 adapters with EcoRI restriction sites and the P2 adapter binding to overhangs generated by NIAIII. Each reaction used 20 μl (200 ng) of digested genomic DNA, 2 μl of T4 ligation buffer (NEB), 5 μl of adapters (0.02 μM Adapter P1 = 0.1 pmol, 3 μM Adapter P2 = 15 pmol), 0.5 μl of T4 ligase (NEB, 200 units), 4 μl ATP 10 mM, and double-distilled water to a final volume of 40 μl. The ligation was performed via polymerase chain reaction (PCR) at 37 °C for 30 min followed by 65 °C for 30 min.

**Table 1:** Sample sequenced using RAD-Seq and summary of RAD –seq analyses

Sample	location	Reads	Number of base pairs	MID	RAD-tag	Mean Depth of RAD-tag	Sequence coverage	Q20 (%)	Q30 (%)
Female									
adC1	Zhejiang	66,274,208	9,531,810,907	GCTAC	6,507,944	10.2	0.19	97.92	93.56
adC2	Zhejiang	139,991,345	20,139,630,928	CCTCT	9,766,767	14.3	0.41	97.94	93.59
adC3	Shandong	155,877,412	22,421,346,688	TAATC	10,029,072	15.54	0.44	97.93	93.58
adC8	Shandong	93,172,870	13,367,981,397	GGCTAC	8,583,073	10.9	0.27	97.91	94.17
Subtotal	–	455,315,835	65,460,769,920	–	34,886,856	50.94	1.31	–	–
Subaverage	–	113,828,958	16,365,192,480	–	8,721,714	12.73	0.33	–	–
Male									
adX1	Zhejiang	127,979,232	18,418,604,178	GCTTA	9,167,503	13.9	0.37	97.97	93.7
adX2	Zhejiang	204,881,740	29,486,474,863	TCCAC	11,078,999	18.5	0.59	97.98	93.7
adX3	Shandong	66,176,492	9,590,086,571	CTCC	6,443,154	10.2	0.20	97.93	93.56
adX8	Shandong	79,719,690	11,421,575,816	ACCTCT	8,038,551	9.9	0.23	97.95	94.1
Subtotal	–	478,757,154	68,916,741,428	–	34,728,207	52.5	1.02	–	–
Subaverage	–	119,689,288	17,229,185,357	–	8,682,051	13.13	0.26	–	–
Total	–	934,072,989	134,377,511,348	–	69,615,063	103.44	2.33	–	–
Average	–	116,759,123	16,797,188,918	–	8,701,882	12.93	0.29	–	–

Following ligation to the adapters, DNA samples were cleaned using the QIAGEN QIAquick PCR Purification Kit, and PCR was carried out to replicate the cleaned fragments. Each PCR reaction contained 10 μl of purified fragments, 10 μl of 5 x NEB Master Mix, 2 μl of primer (10 μm Illumina PE), and 28 μl of double-distilled water to a final volume of 50 μl. PCR conditions were 30 s at 95 °C followed by 16 cycles of 30 s at 95 °C, 20 s at 62 °C, 30 s at 68 °C, and 5 min extension at 72 °C and stored at 4 °C forever. Gel electrophoresis was performed, and fragments ranging from 400 to 600 bp including the 75 bp adapter were excised from the gel, and QIAGEN QIAquick PCR Purification Kit was used to clean the fragments. The samples were sequenced on an Illumina HiSeq PE150 using 150 bp paired-end reads. The sequence was deposited in the NCBI databank (SRP159124).

### Determining sex-specific markers

**Fig. 1:**
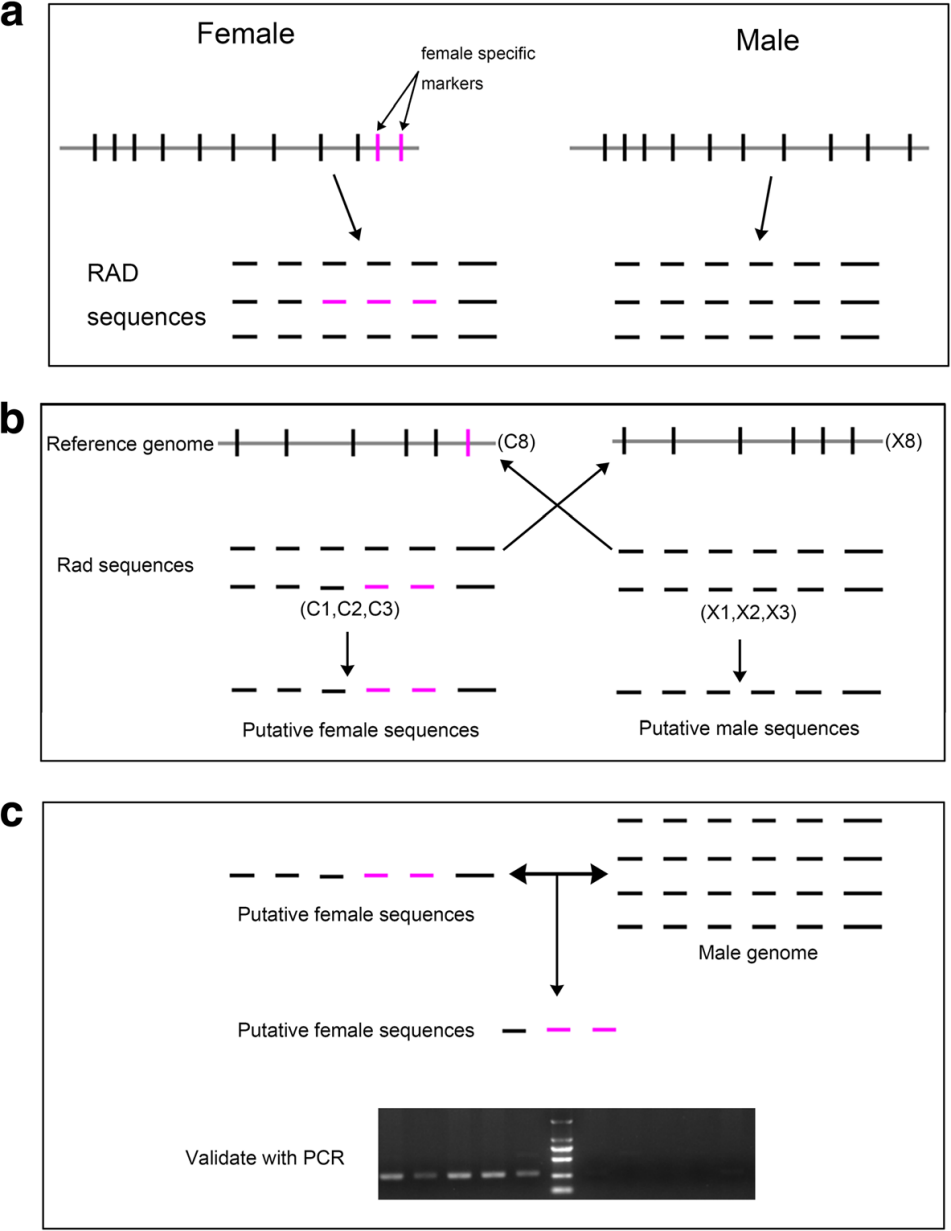
workflow of the female specific marker identification: **a**. locations of restriction sites along chromosomes in male and female specimens and RAD sequences; **b**. bioinformatic analysis of the RADseq libraries; **c**. further analysis of the putative specific marker and validation with PCR

Reads containing low quality sequence scores, adaptor sequences, missing restriction site sequences, and reads with > 10% unknown bases were removed using Trimmomatic v. 0.32 [31], and raw reads were trimmed to 110 nucleotides, which ensured that more than 98% of the nucleotides had a quality value greater than Q30. The generated reads were sorted into loci using Stacks software [32]. The reads from randomly selected individuals, X8 and C8, were assembled as a reference genome by SoapDenovo2 [33]. The assembled sequences were screened against opposite sex raw reads with Stacks v.1.46 [32, 34] (http://catchenlab.life.illinois.edu/stacks/) using the MID sequence to identify exact matches to the110 bp trimmed RAD-tag. The putative female sequences and the male sequences were checked against the male genomic data (incomplete genome data). The unmapped sequence from the female data files was regarded as the candidate female-specific marker (Fig. 1).

### Validation and application of the sex-specific marker

Gonads of all specimens were fixed in 4% paraformaldehyde (pH 7.5) for 24 h, stored in 70% ethanol, dehydrated through an ethanol gradient, cleared in xylene, embedded in paraffin, and 5–6 μm sections were cut and stained with hematoxylin-eosin. The phenotypic sex was determined under light microscopy (Olympus). The genomic DNA was extracted by a DNA extraction kit (TianGen, Beijin, China) and the quality evaluated by agarose gel electrophoresis and NanoPhotometer-N50 (Implen, Germany). One-hundred pairs of primers were designed according to the sequences of the putative female-specific markers by Primer Premier 3.0 (Addtional file 2: Table S1). Polymerase chain reaction reaction was carried out on 7.5 μl 2 × PCR Mix (Dongsheng Biotech, Guangdong, China), 0.2 μl 10 uM forward/reverse primer, 1 μl (50–100 ng/μl) genomic DNA, and doubled-distilled H_2_O to a volume of 15 μl. Reaction conditions were 94 °C for 5 min followed by 35 cycles of 94 °C for 30 s, primer-specific temperature for 30 s, and 72 °C for 40 s with a final extension at 72 °C for 5 min. The putative female-specific markers were initially validated on five male and five female individuals, and further validation was performed on 24 males and 24 females. From a previous study, we collected 12 females and 12 males after high temperature (28 °C) exposure and 20 females and 3 males after 17β-estradiol exposure at concentration of 500 μg/l. The genetic sex was determined by the validated sex-specific marker.

## Results

### Restriction site-associated DNA sequencing and assembling

The RAD-Seq of four male and four female *A. davidianus* was performed on an Illumina HiSeq PE150 sequencing platform, and 93.4 million reads and 134,377,511,348 bp of data were generated. The male RAD-seq library contained 47.8 million reads with 68,916,741,428 bp of data, and the female RAD-seq library produced 45.5 million reads with 65,460,769,920 bp of data, a mean of 11.6 million reads and 16,797,188,918 bp for each salamander. A total of 69,615,063 RAD-tags was used in the study (34,886,856 female and 34,728,207 male) with a mean of 8,701,882 per salamander. The number of the RAD-tags recovered from each specimen ranged from 6,443,154 to 11,078,999 and strongly correlated with the reads recovered from each individual, 66,176,492 to 204,881,740 bp (Table 1). The mean depth of the RAD-tag ranged from 10.2 to 15.54 in female groups and from 9.9 to 18.5 in male groups (Table 1). According to the genome size, the sequence coverage ranged from 0.19–0.44 x in female groups and 0.20–0.59 x in male groups. The average sequence coverage was 0.29 x per individual.

A total of 2,663,744 scaffolds in X8 and 2,949,503 in C8 were assembled with 453,211,396 bp and 584,940,914 bp, respectively. The greatest lengths were 1572 bp in X8 and 1987 bp in C8, and the number of scaffolds > 1000 bp was 67 in X8 and 176 in C8. The N50 length was 148 bp in X8 and 144 bp in C8 (Table 2).

**Table 2:** Summary of the results for the assembly

ID	X8	C8
Scaffold Number	2,663,744	2,949,503
Large scaffolds (> 1000 bps)	65	176
Greatest length (bp)	1572	1987
N50 length (bp)	148	144
N90 length (bp)	144	144
GC content (%)	46.38	46.46
Total base pairs (bp)	453,211,396	584,940,914

### Validation of the sex-specific markers

**Fig. 2:**
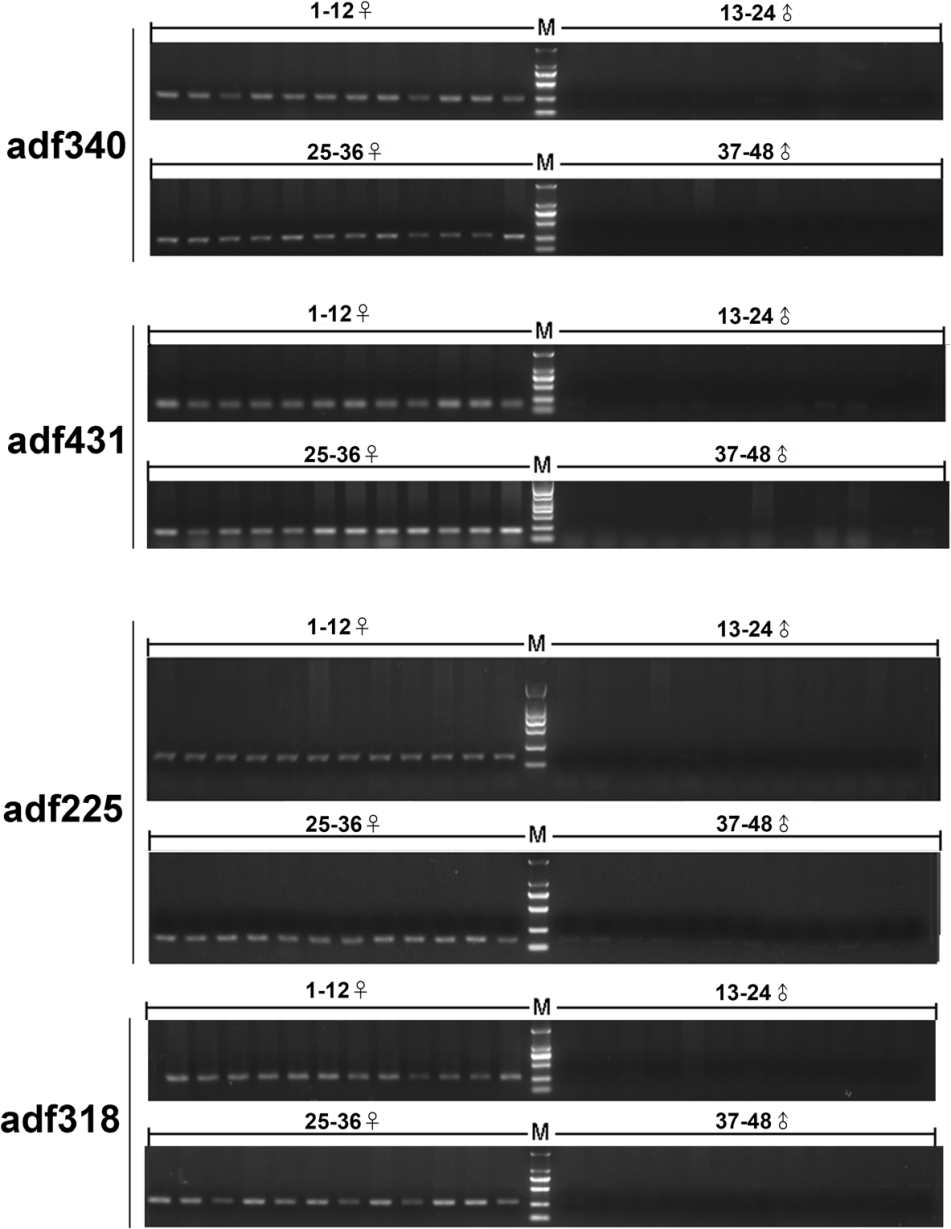
Validation of four female-specific markers in 24 female and 24 male *Andrias davidianus*

Phenotypic sex was determined by examination of gonads (Additional file 1). Female assembled sequences screened against the raw reads of male files (X1, X2, X3) yielded 19,097 female sequences, and 17,994 male sequences after the male assembled sequences for the female raw read files (C1, C2, C3). A further BLAST search was carried out between unmatched sequences and the male genome. Three-hundred-eight female sequences and 542 male sequences were unmapped to the male genome. One-hundred unmapped female sequences were randomly selected, primers were designed according the sequences (Additional file 2), and four female sequences were filtered out for initial validation (Table 3). We defined sequences according to their length as adf431, adf340, adf318, and adf225. The four female sequences exhibited four specific bands in the 24 female specimens and no band in the male (Fig. 2). BLAST searches of the four female-specific sequences in GenBank revealed no high homology sequence.

**Table 3:** Female-specific primers

Primer	Sequence (5′-3′)	Annealing temperature (°C)	Product size (bp)
adf225s	CCATGCCCTGTACATTTGCG	59.899	162
adf225a	CCGTGAACATGGAGGGGTTT	60.251	
adf340s	TTAACGGCCCTAACACCAGG	59.674	251
adf340a	GGTTTAGGGCGGCTCTGATT	60.107	
adf318a	TATGTCAGGGTGATCAAACTCTTCA	59.5	266
adf318s	CTAGAAGACGTGGTGGCCATG	60.0	
adf431a	TCCAGAATGAAGTCCTGGCCT	59.1	178
adf431s	CGAGCCTCCATTGTGCCTT	59.8

### Application of the sex-specific markers

**Fig. 3:**
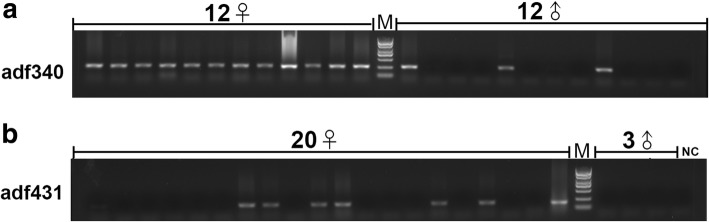
Female-specific marker used to identify sex reversal in *Andrias davidianus* exposed to high temperature or 17β-estradiol. NC, negative control

Twelve males and twelve females exposed to high temperature were examined for the female-specific marker adf340, and all females showed the expected band, while three males displayed a similar band and were identified as genetic female reversed to physiological male (Fig. 3). Twenty females and 3 males exposed to 17β-estradiol were examined by the female-specific marker adf431. Three males and one negative control showed no specific band, and 13 of 20 females displayed no band and were identified as genetic male reversed to physiological female (Fig. 3).

## Discussion

In the recent years, RAD-seq technology was widely used to develop the molecular marker in species. In eight salamanders, 134.38 Gb of data were generated by RAD-seq with mean data per salamander of 16.79 Gb. In *Pistacia vera,* 36.96 Gb of data was sequenced from 18 plants with a mean of 2.05 Gb per plant to identify the sex-linked SNP marker [28]. In *Lupinus angustifolius*, 17.33 Gb data from 20 plants produced 0.87 Gb per plant [35, 36]. In *Anolis carolinensis,* 19.9 million reads generated by RAD-seq and 51, 438 RAD-tag from 17 individuals, recovering RAD-tag per individual from 23,524 to 44,408 [27]. We obtained higher total and mean data per specimen than reported in *Anolis carolinensis, Lupinus angustifolius*, or *Pistacia vera.* Due to the large genome of *A. davidianus* (~50Gb) analyzed by flow cytometry [30], the sequence coverage in this study averaged 0.29 x per salamander. The results were available to explore the sex-specific marker to identify the genetic sex of *A. davidianus,* especially the larvae and sex reversed individuals in the non-model species.

This study was one of few to use RAD-seq to develop sex-specific markers without a genetic linkage map. In *Anolis carolinensis,* sex-specific molecular markers were explored using RAD-seq in seven males and ten females, the accuracy was tested by PCR, and male heterogamety was further confirmed by the sex specific marker [27]. A study of *Pistacia vera* identified sex-linked SNP markers, and the sex determination mechanism was identified as the ZZ/ZW type using the RAD-seq [28]. In other species, the sex-linked marker has been explored using the genetic linkage map by RAD-seq. In Nile tilapia *Oreochromis niloticus*, RAD-seq was used to compare the SNP marker in pseudomales and females, and association analysis with a set of SNPs confirmed that the genomic region of LG23 exerts a significant effect on temperature-dependent sex [37]. In *Polyprion oxygeneios*, a genetic linked group was constructed, and a single major sex-determining locus was mapped to LG 14. Several markers were found to be strongly linked to the sex-determining locus [38]. In *Gadus morhua,* male-specific region of 9 kb was mapped on linkage group 11 annotating a single gene named zkY on the Y chromosome. Expression of zkY was high level in the developing larvae before the onset of sex differentiation [39]. In *Rana clamitans*, 13 sex-linked SNP loci and eight loci associated with males were identified by Diversity Arrays Technology [40, 41], which employs a combination of genome complexity reduction and next-generation sequencing similar to RAD-seq and genotyping-by-sequencing methods [42]. RAD-seq is an effective means of defining a molecular marker.

Using sex-specific sequences to identify genetic sex or the sex chromosome shows advantages over cytogenetics. Cytogenetic methods are not possible in species possessing microchromosomes or lacking a heteromorphic sex chromosome, such as the majority of amphibians, reptiles, and fish [19, 43–45]. *A. davidianus* has been reported to possess 30 pairs of chromosomes including 19 pairs of telocentric chromosomes [18], enabling identification of a sex-specific marker.

We isolated the female-specific marker and used it to reveal the genetic sex. We identified three males reversed from genetic females exposed to high temperature and 13 females reversed from genetic males exposed to 17β-estradiol. These results agreed with our previous study in which sex reversed individuals were identified by a different sex-specific marker [46], suggesting that these sex specific-markers are highly consistent and valid. In *Pelteobagrus fulvidraco*, Y- and X-linked markers were isolated and used to identify sex reversed individuals and YY super-males to produce the all-male population [3]. In *Pseudobagrus ussuriensis*, male-specific sequences were employed to identify genetic sex, suggesting male heterogametic sex determination [47]. A sex-specific sequence was used to identify genetic sex and the WW super female in *Cynoglossus semilaevis* [20, 21]. A sex-specific marker was used to genotype *Hyla arborea* and showed that all females were homozygous for allele 235, while the males were heterozygous (235/241), suggesting a male heterogamety sex determination system [48].

Results of the present study have implications for both fundamental and applied research: First, the sex-specific marker can be used to identify the sex determination system, which will help describe the evolution of sex determination in amphibians. Second, the sex specific marker allows investigation of parent influence on offspring sex ratio. Third, identification of the sex-specific marker has potential value for conservation biology. Climate change has been shown to affect the sex ratio of the green sea turtle *Chelonia mydas*, in the Southwest Pacific, and warm northern Great Barrier Reef nesting beaches were female-biased at 99.1% of juveniles, 99.8% of subadults, and 86.8% of adults [49]. In order to protect *C. mydas* from extinction due to complete feminization, male specimens were identified and released. Similarly, the *A. davidianus* sex specific marker could allow release into the wild at the optimum sex ratio.

## Additional files

Additional file 1: Histology sections of the gonads of *Andrias davidianus*. Female gonad; B. Male gonad. GrC: Granulosa cells; GC: Germ cell; FC: Follicular cavity; SL: seminiferous lobule; SC: somatic cell. (TIF 7268 kb)

Additional file 2: Primers used. (DOCX 35 kb)

## Abbreviations

AFLP: Amplified fragment length polymorphism
Chr-4: Chromosome 4
LG: Link group
PCR: Polymerase chain reaction
RAD: Restriction site-associated DNA sequencing
SNP: Single nucleotide polymorphisms

## Authors’ information

Q.M. Hu., Q.H.W., H.F.T., Y.M., H.B.X., Yangtze River Fisheries Research Institute, Chinese Academy of Fishery Sciences, Wuhan, Hubei 430223, China; C.F. C., C.S.X., State Key Laboratory Cultivation Base for Cell Differentiation Regulation, College of Life Science, Henan Normal University, Xinxiang 453007, Henan Province, China; Q.H.W., College of Life Science, Yangtze University, Jingzhou 434025, China; Z.G.Q., L.W., College of Fisheries, Henan Normal University, Xinxiang 453007, China.
