# Supplementary figures and images for: Genome-wide RAD sequencing to identify a sex-specific marker in Chinese giant salamander Andrias davidianus
Source: BMC Genomics. 2019 May 23;20:415. doi: 10.1186/s12864-019-5771-5 (PMC6533744; doi:10.1186/s12864-019-5771-5)

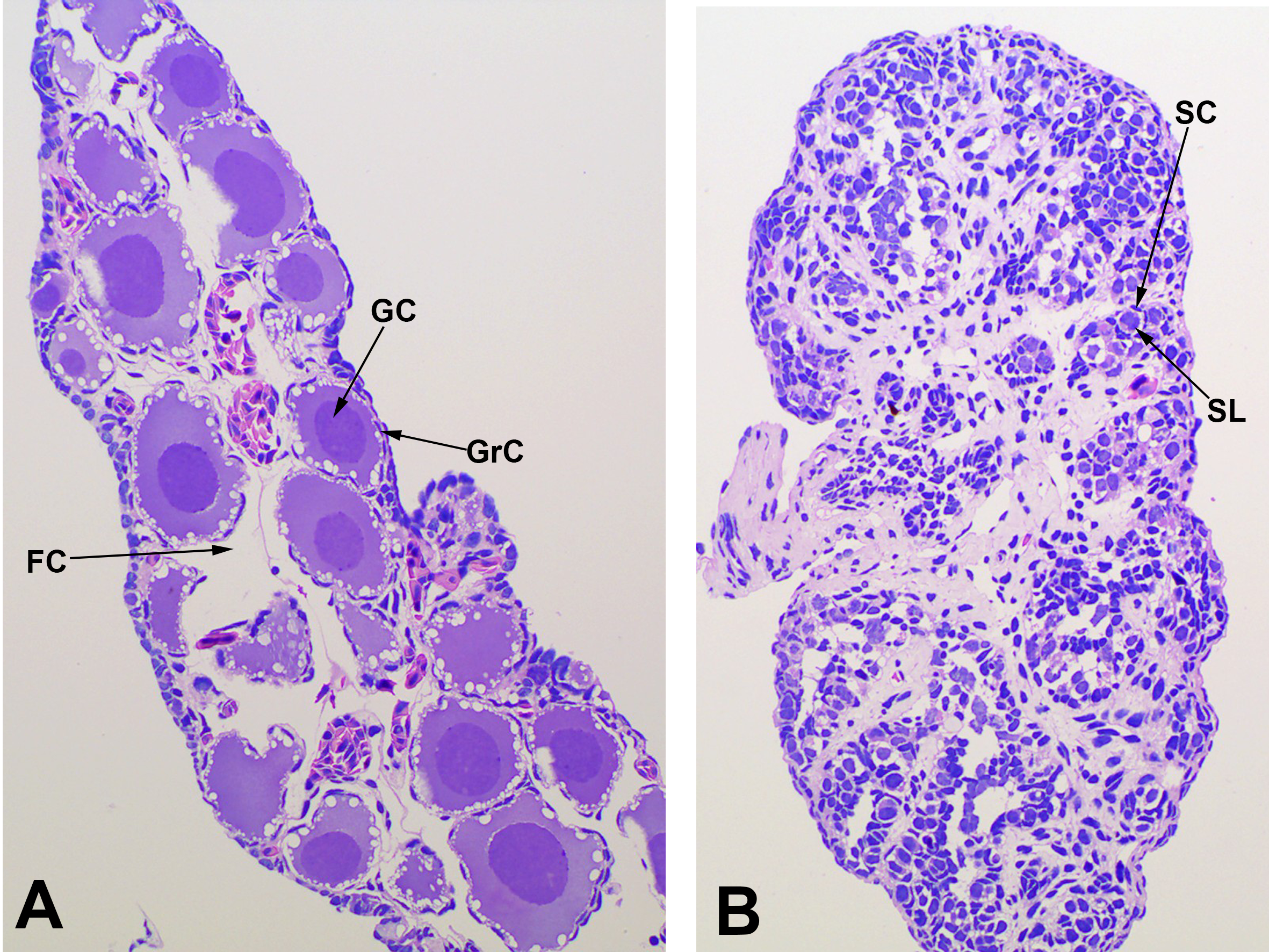

Supplement: Supplementary file 1 — Histology sections of the gonads of Andrias davidianus. Female gonad; B. Male gonad. GrC: Granulosa cells; GC: Germ cell; FC: Follicular cavity; SL: seminiferous lobule; SC: somatic cell. (TIF 7268 kb) [file 12864_2019_5771_MOESM1_ESM.tif]
