# Supplementary material for: Genome-wide RAD sequencing to identify a sex-specific marker in Chinese giant salamander Andrias davidianus
Source: BMC Genomics. 2019 May 23;20:415. doi: 10.1186/s12864-019-5771-5 (PMC6533744; doi:10.1186/s12864-019-5771-5)
Supplement: Supplementary file 2 — Primers used. (DOCX 35 kb) [file 12864_2019_5771_MOESM2_ESM.docx]

**Additional file 2:** Primers used

| Primer | Sequence (5’-3’) | Annealing temperature（℃） | Product size (bp) |
| --- | --- | --- | --- |
| C8_6014380s | GAAGGCCGTCAGAAAGCCTA | 59.751 | 201 |
| C8_6014380a | ATCCTGCATCACGACCCATC | 59.893 |  |
| C8_6942106s | GACGAGGTCCACCGTGAAG | 60.081 | 232 |
| C8_6942106a | GGTCTGAACCCGGCAAACTA | 59.964 |  |
| C8_8123623s | CGGGTCCGGAAATATGTTGC | 59.339 | 241 |
| C8_8123623a | ATTCCCGTCATGCATCAGGG | 60.179 |  |
| **C8_4055433s** | **CCATGCCCTGTACATTTGCG** | 59.899 | 162 |
| **C8_4055433a** | **CCGTGAACATGGAGGGGTTT** | 60.251 |  |
| C8_7174324s | TGGAACGACTTCGCCTATGG | 59.825 | 203 |
| C8_7174324a | GGCGCAGGATCGGGTATTTA | 59.966 |  |
| C8_3433188s | CAGCACGGAAAGTAGGTCCA | 59.68 | 238 |
| C8_3433188a | TGAGCAGGCGCACTTAAGAT | 59.75 |  |
| C8_5758217s | ATTCCATAGCCATAGCCCGC | 60.035 | 206 |
| C8_5758217a | CTGGGATTGTGGTCCGGC | 60.438 |  |
| C8_7611277s | GCGCGTACTACCGAATCTCA | 59.971 | 202 |
| C8_7611277a | GCCACATGCTCAGCTGAGTA | 60.108 |  |
| C8_8123677s | TTTCGAAGGGTTGGAGCCTG | 60.251 | 216 |
| C8_8123677a | AAGCGCATTGGACACCCAG | 60.973 |  |
| C8_6746939s | TGCAATCCAAGCTTCATTTCCG | 60.095 | 210 |
| C8_6746939a | TGAACGCAGAATCAGACCCA | 59.315 |  |
| C8_4183121s | ATGCCCTCCCATGTAACGTG | 60.107 | 200 |
| C8_4183121a | TGGAATCTATTGAAGCATTGCTGA | 58.805 |  |
| C8_7021199s | TGGAAACGCTCTATTGCTTTT | 56.44 | 209 |
| C8_7021199a | CCAATTCTTCTAAAAATTGCTCCGG | 59.193 |  |
| C8_8130535s | AATGCCAAGGAAACGCCATC | 59.468 | 273 |
| C8_8130535a | TCTCCGGATTGGCCTTTCAG | 59.747 |  |
| C8_7473066s | AAATCTGGTGGAGAAGCGGC | 60.678 | 253 |
| C8_7473066a | AATTCCTCGCCGCCTATTCG | 60.6 |  |
| C8_7323045s | CCCTTGAACTCGAGCACCAA | 60.25 | 207 |
| C8_7323045a | GAGTGCCTGGTGGAGGAC | 59.334 |  |
| C8_6976931s | CTCTGCGTACACGTATGCCT | 59.899 | 250 |
| C8_6976931a | CACCAAGCTTGAGGATTACCA | 57.928 |  |
| C8_8061156s | GGTCTTGGAGTCTTCGGTCG | 60.109 | 222 |
| C8_8061156a | TCCGCATCGAACAGCTCTAC | 59.899 |  |
| C8_6280462s | GGAATAACTTTCCGAACTTCAAAAACC | 59.831 | 200 |
| C8_6280462a | AGGTCCGCATTACTTATGGTGT | 59.496 |  |
| C8_6869387s | CTGACGGTCTTGGACTGGTC | 60.038 | 238 |
| C8_6869387a | TCGCCGTTATCCAGTTTGCT | 60.037 |  |
| C8_5693092s | TCAACGTCATGCCCGAAGAA | 59.966 | 219 |
| C8_5693092a | GAGATCATGCGCCTGTACGA | 59.969 |  |
| C8_8117363s | ACAAGGTTACCGTCCAGCTG | 59.965 | 243 |
| C8_8117363a | TCCTTGCAAATAACGCGCAC | 60.11 |  |
| C8_7287631s | TGGCAATTATGTGCTCGTTGA | 58.569 | 229 |
| C8_7287631a | GGCTGTGGGTGTCTTCTGAA | 59.891 |  |
| C8_7363817s | AATTCGGTGCCCTTCTGGAG | 60.035 | 254 |
| C8_7363817a | CGGTTCCGTTGGTCGTGA | 59.972 |  |
| C8_5905986s | TTAAAGCGCTACGGCAATGG | 59.27 | 206 |
| C8_5905986a | CTGGGGCTGATCTTCACCTT | 59.377 |  |
| C8_6367646s | GCATGGCTTTATGGAGGGGT | 60.106 | 202 |
| C8_6367646a | CTGTTTCCCAGATGTGCTCCT | 59.996 |  |
| C8_5800342s | TGAAGATCCGTTGAGAGCCG | 59.827 | 201 |
| C8_5800342a | TCCGGAAACCCATGAATTAAAAGC | 60.082 |  |
| C8_7377011s | CAACAATGACCAGCACCTGC | 60.039 | 222 |
| C8_7377011a | CTTGGGCGGGACCTTGAG | 60.046 |  |
| C8_3910877s | TCTGGTATTGATCAAAAATTGGCA | 57.398 | 203 |
| C8_3910877a | ATGGCGTCTAACCATGTCCC | 59.82 |  |
| C8_7986068s | TGTTGGCAGCATTTTCAGCA | 59.249 | 227 |
| C8_7986068a | AGATCCGAATACTGCTGCCG | 59.967 |  |
| C8_4747111s | TCCCCAACATGGAGCATCAC | 60.034 | 205 |
| C8_4747111a | GCCCCTCTCAGCGATTAGAC | 59.967 |  |
| C8_8090657s | TCATTTCAGTTCTGTCAGAATCTGA | 58.238 | 213 |
| C8_8090657a | GGAGCAGTTCCAAACCCGAT | 60.323 |  |
| C8_5612669s | GCAGATGTTGATTTCCGGGC | 59.899 | 201 |
| C8_5612669a | CAACATAGCCGGCCTCTGTT | 60.393 |  |
| C8_7275786s | TGAAATCGGCGTCGGGAATG | 61.087 | 210 |
| C8_7275786a | AAGAAGTCGAGACCTGCGAC | 59.759 |  |
| C8_8161239s | GGACCGGCTCCTTTTACCAT | 59.745 | 204 |
| C8_8161239a | ATAGCGTTCGCTCACTCCTG | 59.899 |  |
| C8_6410436s | AGCATCCCATTATGGAACGCT | 59.856 | 201 |
| C8_6410436a | TTGCCCTGATTTCACTCAGA | 56.749 |  |
| C8_6060920s | AACTGTGCCAGTAGTCAGGC | 59.965 | 215 |
| C8_6060920a | TGAATCCCCTTCAGCGTTCC | 60.035 |  |
| C8_5186561s | TCGCGGGGTTACGAATACTG | 59.899 | 218 |
| C8_5186561a | GCCGACAAATTTCGCCATCT | 59.548 |  |
| C8_5615262s | TCGTTATGATCAGGCATCTACT | 56.522 | 222 |
| C8_5615262a | GCACCTGTTGCCCTTCAGAT | 60.61 |  |
| C8_7955072s | TGAACCGTATGAAATGCTGGA | 57.658 | 238 |
| C8_7955072a | CCCAGGTCTGTAAGAGCCGA | 60.971 |  |
| C8_6552549s | AATTCCAGAACACCAGCGCC | 61.244 | 248 |
| C8_6552549a | ATTTCCACCCGGCTGTTCC | 60.304 |  |
| C8_5909132s | TCCGTAGTCGTCGAGATGTTC | 59.337 | 206 |
| C8_5909132a | GCGGTGATCGGTGAACCTAA | 60.109 |  |
| C8_4254413s | TTGCAACTTCAATGGCTGCA | 59.249 | 231 |
| C8_4254413a | TCATGCTGGAAACTGGTCCT | 58.933 |  |
| C8_6924790s | CCCGACCTCAACCTGGATC | 59.477 | 200 |
| C8_6924790a | TCACGGAAGCCTGGTTGAAG | 60.25 |  |
| C8_7780380s | GCTCCAAAGGTGAGCCATGA | 60.323 | 218 |
| C8_7780380a | AGCAGTGTGAGGAAAGACTAA | 56.595 |  |
| C8_5907267s | AATTCACCGCCACCAAGGTC | 60.894 | 221 |
| C8_5907267a | CGGTGTCGACGGTTTTGC | 59.753 |  |
| C8_6942829s | GTAGTCGTCCACCGCATTCC | 60.805 | 202 |
| C8_6942829a | CTCGGGCTGTTCGACGAC | 60.504 |  |
| C8_5684842s | CAACTGCGTCAGCTCTACGA | 60.11 | 206 |
| C8_5684842a | TTCGCGGTCGTAGCTGAAAT | 60.109 |  |
| C8_6198233s | TGTACTGAGCACTGCTTCAGG | 59.999 | 200 |
| C8_6198233a | ACAGATTTAAGGGGCTGGGC | 60.033 |  |
| **C8_728945s** | **TTAACGGCCCTAACACCAGG** | 59.674 | 251 |
| **C8_728945a** | **GGTTTAGGGCGGCTCTGATT** | 60.107 |  |
| C8_3664528s | TGATCCCTAAAAAGAAGGATGCT | 57.543 | 209 |
| C8_3664528a | ACCTAACAAACCTTTTCTAGCAGG | 58.929 |  |
| C8_4914021s | GCTCATCCTCGGCTTTGACT | 60.108 | 266 |
| C8_4914021a | CCTACTTGCGCGAACTGTTG | 59.836 |  |
| C8_5264511s | GTCGCCTGATCGAGTACCAG | 59.97 | 231 |
| C8_5264511a | GTCGGCCTTGATGCATTCAC | 59.901 |  |
| C8_5454881s | ACCCACGTATGCATTCAAACT | 58.21 | 207 |
| C8_5454881a | ACCCAAATTAAGTCTGAAAGATCA | 56.274 |  |
| C8_5931871s | TCCTCTACGCTTCCAGTCCA | 59.961 | 230 |
| C8_5931871a | CAACGGCCTAGACTTTGGCT | 60.322 |  |
| C8_7156187a | CCTTCATCGGCAGTAACCTGA | 59.793 | 247 |
| C8_7156187s | ATGTCAACCGGAGTTCTCGT | 59.033 |  |
| **C8_5894218a** | **TATGTCAGGGTGATCAAACTCTTCA** | 59.5 | 266 |
| **C8_5894218s** | **CTAGAAGACGTGGTGGCCATG** | 60 |  |
| C8_3849262a | TCAAGCAAGGAGACCAGCTC | 59.677 | 266 |
| C8_3849262s | AGCCAATGGATCACTGGCAA | 59.96 |  |
| C8_4135419a | GCGGCATGTGCAAGATACAA | 59.549 | 201 |
| C8_4135419s | TCGTAGCCTTCCATTTCCGG | 59.823 |  |
| C8_7737978a | ATTCTCTGGCAACGATCGCA | 60.108 | 228 |
| C8_7737978s | TACAGCGTGGACGGATCTTG | 59.827 |  |
| C8_5928363a | AATTCACGCACAGCTTTCGC | 60.386 | 232 |
| C8_5928363s | TTGCTGCGCCAAATGATTCC | 60.109 |  |
| C8_6290354a | GCGATACCCCACAACCACTT | 60.322 | 223 |
| C8_6290354s | CTGGGTTTCGGTTCCGGTC | 60.67 |  |
| C8_5751679a | TCATCGTTGACCCTGAAGCC | 60.036 | 245 |
| C8_5751679s | ACCCTTCTCGATCGACGTTG | 59.829 |  |
| C8_3957607a | CTTGCTGGAATCCGCTCTCT | 59.822 | 312 |
| C8_3957607s | ATGCACCTTCATGAGTGGCA | 59.961 |  |
| C8_6293175a | CAACTGGCTAGCCTCTCCAC | 60.108 | 223 |
| C8_6293175s | TCTTTCACGGCCTCAGGTTC | 59.965 |  |
| C8_6293455a | TTGCAAAAAGACACCGTGCG | 60.524 | 200 |
| C8_6293455s | AGGCTCAATTCCTGGAGAAGTC | 59.762 |  |
| C8_6285951a | GTCGCCAATCTGGACGTGTA | 60.109 | 204 |
| C8_6285951s | CAAATGCCTGCGCTCGTATC | 60.042 |  |
| C8_7274649a | GATTCGCTCTCGGCCCTG | 60.28 | 214 |
| C8_7274649s | CACGCTCTCCACATCCTGG | 60.153 |  |
| C8_8187467a | ACCACCGTTTACAGCACAGT | 59.821 | 225 |
| C8_8187467s | GGGCACATTAGCAGCTCAGA | 60.108 |  |
| C8_6247604a | TGATGTCTTTCTCTGGCTAGCA | 59.166 | 223 |
| C8_6247604s | ACGAGCATGGTCATCATAGCA | 59.586 |  |
| C8_7769807a | ACTGGAGTTAATTATCAAGGAGTTCA | 58.082 | 230 |
| C8_7769807s | TCAATTGTTTGCTGCTGCCC | 59.966 |  |
| C8_5587993a | TGTGCCTTCATCATAGCAGGG | 60.134 | 245 |
| C8_5587993s | CCGAATGCAGAGGGGAAAGT | 60.035 |  |
| C8_7226554a | TCTCCTTCTACACCTAAATACCCA | 58.154 | 212 |
| C8_7226554s | ATCTTCAGGCCATTGCGACA | 60.035 |  |
| C8_7290977a | ATTCGCTCCCACAGTGATCG | 60.179 | 218 |
| C8_7290977s | CCGATCCGGTTGCAGATACA | 59.896 |  |
| C8_8249657a | TCAAGAGGAATAAACTTGGCGC | 59.253 | 200 |
| C8_8249657s | ACCGCTTTTCCTACTCCTTCA | 59.024 |  |
| C8_7322414a | AATTCGGCCTGAGTCTGCG | 60.449 | 210 |
| C8_7322414s | CAACCCATCTGCACCACCTC | 60.962 |  |
| C8_5754421a | TCCCTTTCAATTTTGGCCGC | 59.68 | 210 |
| C8_5754421s | CAAACGGTCGAGCAAGTTCG | 60.111 |  |
| C8_2647361a | GAGAGCGGGCATATCAGCG | 60.736 | 235 |
| C8_2647361s | CATGCTCGCTGGAATGCTG | 59.644 |  |
| C8_6553066a | ATTCCAGAACCGCAACTCCC | 60.323 | 215 |
| C8_6553066s | ACCAGAGTAAAGGCATGGGC | 60.034 |  |
| C8_7833710a | AATTCTGCAGGACTGGAGCC | 60.035 | 200 |
| C8_7833710s | TTCCAACTGATTGATTTCGTCGT | 58.938 |  |
| **C8_4433699a** | **TCCAGAATGAAGTCCTGGCCT** | 59.1 | 178 |
| **C8_4433699s** | **CGAGCCTCCATTGTGCCTT** | 59.8 |  |
| C8_6821305a | GAGTGCCGAATCATCAGGCT | 60.179 | 258 |
| C8_6821305s | GCTCTGGTTGGCATTCCTGA | 60.323 |  |
| C8_5719884a | TCGAAAGACTTAGGCAGCCG | 60.109 | 228 |
| C8_5719884s | TGACCTGATCGTCGGCAAAA | 59.966 |  |
| C8_6928918a | ACACCTACGGCAAAGTCGAG | 60.039 | 200 |
| C8_6928918s | GTCGAGCACTCCTTGTACCC | 60.109 |  |
| C8_7597206a | AGGGGTATAGGCCTGTACCG | 60.179 | 204 |
| C8_7597206s | ATTGCAGGTGGGCTGAATCA | 59.96 |  |
| C8_7139021a | AAGGTCTGGCTGGATGCATT | 59.666 | 220 |
| C8_7139021s | TGGAGATCCGGTATCCAAGGT | 60.062 |  |
| C8_8140032a | TCTGGTGAAGAGTGCCTGTG | 59.606 | 226 |
| C8_8140032s | GCAGTGGCTGAATGGACTGA | 60.322 |  |
| C8_7305121a | TGGGGATCTCTGTGGGTGAT | 59.955 | 212 |
| C8_7305121s | TGCAACCCCAATAATGGTGA | 57.388 |  |
| C8_6090505a | GGTATCGACACCCAGTTGCT | 59.751 | 249 |
| C8_6090505s | CAGATCGGTCTTGCTCACCA | 59.753 |  |
| C8_7006452a | TCACATAGCACTGAAGGGTCT | 58.46 | 232 |
| C8_7006452s | TGGGCATTCATGTAGAGATTTGT | 58.141 |  |
| C8_7246357a | ACGATCGATTGGAATGCCGA | 59.896 | 212 |
| C8_7246357s | CTTGATCGGGGAGTTCTGCC | 60.463 |  |
| C8_7671819a | AATTCTCCGATCTCGTGGCG | 60.249 | 235 |
| C8_7671819s | GAAGGAGGAGCTTCCGGAAC | 60.108 |  |
| C8_7402136a | CGTTCTGATGGACAGGGTCG | 60.459 | 203 |
| C8_7402136s | GCCTTGGCTGACTCGATGAT | 60.179 |  |
| C8_7402044a | ATTCGTTCTCAGAGACCGCC | 59.826 | 255 |
| C8_7402044s | GACACCGGCTAATTGGTCGA | 60.109 |  |
| C8_7639388a | GTGTTTCACGTGCACCTGAC | 59.972 | 247 |
| C8_7639388s | TACAGCCCCTTCCGTCTCAA | 60.543 |  |
| C8_6276103a | CTTTTCCTGCCAGATCGCCT | 60.394 | 205 |
| C8_6276103s | ACACGTCGTGAAGATCTGGC | 60.389 |  |
| C8_7357485a | GGTAAGGCCTTTCGGTCGAG | 60.46 | 252 |
| C8_7357485s | GACTTCCAGGTCAAGGTCCG | 60.037 |  |
| C8_7543181a | TCTCCGATTATCTGGTGGAAACA | 59.227 | 203 |
| C8_7543181s | AGAATCACTTCATTAGCTTCCAAAGC | 60.128 |  |
| C8_2746007a | CCTGAGCATCCGTCAAACCT | 60.036 | 245 |
| C8_2746007s | TGCATTGAAGCAGAATCCTACT | 57.774 |  |
| C8_6566067a | CACACCCCCTCTATCTCCCA | 60.031 | 222 |
| C8_6566067s | GACTACACCCTGCCCACTTG | 60.322 |  |
| C8_6836847a | GGTTCTTATCTTCCCAGTCTCCA | 59.225 | 205 |
| C8_6836847s | ACTAGCCCAAGTCAAAGAATAGT | 57.236 |  |
